# Impaired Daytime Urinary Sodium Excretion Impacts Nighttime Blood Pressure and Nocturnal Dipping at Older Ages in the General Population

**DOI:** 10.3390/nu12072013

**Published:** 2020-07-07

**Authors:** Rosaria Del Giorno, Chiara Troiani, Sofia Gabutti, Kevyn Stefanelli, Sandro Puggelli, Luca Gabutti

**Affiliations:** 1 Department of Internal Medicine, Clinical Research Unit, Regional Hospital of Bellinzona and Valli, Ente Ospedaliero Cantonale, 6500 Bellinzona, Switzerland; chiara.troiani@students.uniroma2.eu (C.T.); gabuttisofia@gmail.com (S.G.); sandro.puggelli@eoc.ch (S.P.); 2 Institute of Biomedicine, University of Southern Switzerland, 6900 Lugano, Switzerland; 3 Department of Social Sciences and Economics; Sapienza University of Rome, 00185 Rome, Italy; kevyn.stefanelli@uniroma1.it

**Keywords:** urinary sodium excretion, nighttime urinary sodium excretion, daytime urinary sodium excretion, 24-h blood pressure, nocturnal dipping

## Abstract

The circadian rhythm of urinary sodium excretion is related to the diurnal blood pressure regulation (BP) and the nocturnal dipping pattern. The renal sodium excretion expressed as daytime/nighttime ratio impacts BP, but a limited number of studies have investigated this topic to date. In this cross-sectional study, we aimed to investigate the impact of different daily patterns of sodium excretion (comparing low with high ratios) on BP and nocturnal dipping and to explore the relationship with age. Twenty-four-hour ambulatory BP monitoring and daytime and nighttime urinary sodium collections were used to assess 1062 subjects in Switzerland. Analyses were performed according to the day/night urinary sodium excretion ratio quartiles (Q1–Q4) and by age group (≤50 and ≥50 years). Subjects in Q1 can be considered low excretors of sodium during the daytime since the rate of sodium excretion during the daytime was 40% lower than that of subjects in Q4. Quartiles of the day/night urinary sodium excretion ratio showed that subjects in Q1 were 7 years older and had respectively 6 and 5 mmHg higher nighttime systolic and diastolic BP and a higher nocturnal dipping compared with subjects in Q4 (*p*-value ≤0.001). Associations found were significant only for subjects older than 50 years (all *p* < 0.05). The present results suggest that a decreased capacity to excrete sodium during daytime is more prevalent as age increases and that it impacts nighttime blood pressure and nocturnal dipping in older subjects.

## 1. Introduction

Excessive dietary sodium intake represents a pathogenic determinant and a key modifiable environmental risk factor for hypertension [1]. However, despite the long-established evidence of the deleterious effect of high sodium dietary intake on blood pressure (BP), several questions remain unanswered. To date, 24-h urine collection has been recommended as the gold standard for the assessment of salt intake in the population [2]. Nevertheless, many factors, ranging from the mechanisms of sodium absorption to metabolic and environmental factors, can affect urinary sodium excretion [3]. The complex mechanisms underlying renal sodium handling could be involved in the inter-individual variability and in the responses of BP to dietary sodium intake [4,5]. A still open field of investigation into the mechanisms of renal sodium handling is the peculiar daily rhythm of sodium excretion in the urine, typically characterized by the maximum level of excretion during the daytime and the minimum at night [6]. This circadian rhythm of sodium excretion represents an intriguing phenomenon related to blood pressure (BP) diurnal variation and represents a key determinant in the genesis of the BP non-dipping pattern [6,7].

Previous findings have suggested that BP represents the primary determinant of sodium excretion during the night, indicating that an impaired capacity to excrete sodium during the daytime is linked to increased nocturnal BP in order to promote sodium excretion, thus resulting in a typical non-dipping pattern [6,8,9]. Nevertheless, the daytime/nighttime rhythm of urinary sodium excretion has not been extensively investigated, and is still not completely understood.

At present, what is known is that the diurnal pattern of sodium excretion can be reversed or altered by hypertension [10]. The findings of a few studies in a small sample of subjects have demonstrated flattened daytime/nighttime urinary sodium excretion in hypersensitive adults [11].

Several factors could contribute to an impairment in sodium excretion capacity, among which reduced renal function (e.g., subjects with a low glomerular filtration rate), increased tubular sodium reabsorption (e.g., primary aldosteronism) and specific hormonal effects are the most relevant [12].

Moreover, experimental models have shown that the capacity of the kidney to manage sodium intake (conserving or removing, depending on restriction or excess of salt intake) and to produce an adequate pressure natriuresis decreases with aging [13,14]. An impairment in pressure-natriuresis mechanisms leads to salt-sensitive hypertension, which shows a substantially increased incidence as age increases, leading to a higher prevalence of low-renin essential hypertension in older subjects [15].

Previous findings have suggested that an impaired renal capacity of sodium excretion during the daytime could be relevant in determining a BP increase at night, resulting in a non-dipping pattern [11,16].

Nevertheless, studies that support this hypothesis have been mainly conducted in a selected patient population (i.e., salt-sensitive, salt-resistant) or in a larger sample of subjects in selected geographic regions.

Several previous epidemiological and interventional studies showed a dose–response association between BP and sodium intake [17,18]. In this context, the 24-h ambulatory BP has shown the strongest relationships with CV events, and nighttime BP has been shown to have the strongest association with cardiovascular outcomes [19].

However, studies to investigate the difference in daytime/nighttime urinary sodium excretion ratio in the general population and its impact on blood pressure are still lacking.

On the basis of all the above-mentioned concepts, it is conceivable to hypothesize that daytime/nighttime urinary sodium excretion ratio could have a peculiar pattern in the general population and most likely a different impact on ambulatory 24 h BP (ABPM). For this purpose, we analyzed the data of a large sample of subjects in Switzerland, including both normotensive and hypertensive, from whom separate daytime and nighttime urine samples and 24-h ABPM recordings were collected. In the present study, we aimed to investigate the impact of the different daily patterns of sodium excretion (low vs. high daytime excretion) on BP and nocturnal dipping and to assess whether different associations occur according to age.

## 2. Methods

### 2.1. Study Participants

The present analysis is based on data from a population-based cross-sectional study conducted between 2017 and 2018 in Southern Switzerland to explore determinants of arterial stiffness and cardiovascular risk factors of the resident population. The design and methodology have been detailed previously [20]. Briefly, based on the official list of the resident population (Swiss Federal Statistical Department) participants were recruited by a simple random sampling method, based on residence, gender, and age group. Of the individuals asked to participate, 86% agreed. Recruitment of the study population took into consideration the sub-regional residency of the Swiss region under examination (Ticino). To address any geographic differences, a sub-district recruitment, based on the district-area of Canton residency, was also performed. At the end of the recruitment period, a total of 1202 subjects were investigated.

The study was carried out in accordance with the Helsinki Declaration and was approved by the local Swiss ethics committee. All participants provided informed written consent. Data and analyses are presented in accordance with Strengthening the Reporting of Observational Studies in Epidemiology (STROBE) [21]. Participants were of Caucasian origin. We excluded 76 individuals on the basis of incomplete urine collection in either the daytime or nighttime and 64 individuals for incomplete data from ABPM. The present analyses are therefore, based on 1062 individuals, all Caucasian.

### 2.2. Sodium Excretion

All study participants underwent two consecutive visits at the hospital’s research unit of the internal medicine department of the teaching hospital of Bellinzona and Valli (Switzerland).

All individuals were investigated while on their usual diet. Twenty-four hour urine collections were performed on the same day as ABPM. Two separate collections of urine were performed: one for the daytime period and another one for the nighttime. Study participants visited the hospital’s research unit of the internal medicine department at the Regional Hospital of Bellinzona (Switzerland) in the morning and received detailed instructions explaining the study procedure. 

During the first visit, participants were given two containers for 2 L urine collection, marked as “daytime urine” and “nighttime urine”, and a plastic beaker to transfer the urine to the urine collection containers. Daytime and nighttime were defined according to each participant’s self-reported bedtime and wake-up time. A urine collection diary was also provided in which each participant marked (i) the starting diurnal hour of urine collection, (ii) the starting hour of nighttime urine collection and (iii) the hour of the last urine sample collected. All urine during the following 24 h was collected and the first urine of the waking period was added to the night urine collection. The next day, the participants visited the hospital’s research unit a second time and the collected urine containers were transferred to the Biochemistry Laboratory of Ente Ospedaliero Cantonale, Regional Hospital of Bellinzona, for the analyses.

Urinary sodium and potassium concentrations were measured by a non-ion-selective electrode method (measured using a Roche Hitachi; an indirect ion-selective electrode).

Urinary albumin concentration and creatinine were determined using a Behring Nephelometer (Siemens BN albumin; Siemens Healthcare, Marburg, Germany) and a Hitachi 717 device (Roche Diagnostics, Mannheim, Germany), respectively. All urine assessments of the daytime and nighttime periods were performed separately.

The validity of the 24-h urine collection was assessed through a combination of self-reported urine loss and the 24-h urine volume (excluded in cases of insufficient urine collection, i.e., urine volume <500 mL/24 h, a self-reported loss of a urine sample of more than 100  mL or more than one time). 

The amount of excreted 24-h urine sodium and potassium was calculated by multiplying the total volume of the collected urine by the concentration of measured sodium.

### 2.3. Twenty-Four Hour Ambulatory Blood Pressure Monitoring

For each study participant, a device for twenty-four hour ambulatory BP monitoring (ABPM) was programmed to register BP every 30 min during the daytime and every hour during the nighttime. A validated, automated non-invasive oscillometric device (Mobil-O-Graph, I.E.M. GmbH, Stolberg, Germany) was used. BP recordings were performed on working days, and the patients were instructed to maintain their usual activities. Appropriate cuff sizes were used. The 24-h ambulatory BP was recorded simultaneously with the collection of 24-h urine. Hypertension was defined on the basis of a previously reported personal diagnosis of hypertension or the current use of antihypertensive medication with a previous diagnosis of hypertension. All BP parameters provided by ABPM were recorded. Daytime and nighttime periods were pre-defined. Systolic blood pressure (SBP), diastolic blood pressure (DBP), mean blood pressure (MBP) and pulse pressure (PP) were recorded for the daytime and nighttime periods.

### 2.4. Assessment of Other Study Variables

During the first visit, a medical examination and a standardized interview were performed in which information on socio-demographic characteristics, comorbidities and traditional cardiovascular risk factors (personal and familial) were collected. A detailed drug anamnesis (generic drug names with the commercial one, dose, treatment duration, current and previous medication) was also performed. The following parameters were assessed: height (cm), weight (kg), waist circumference (cm), hip circumference (cm), body mass index (BMI; kg/m^2^) and neck circumference (cm). Blood pressure (BP) in the office was measured during the first examination using a validated automatic oscillometric device following standardized procedures (Dinamap model Pro 100 automated oscillometric sphygmomanometer, Critikon, Tampa, FL, USA).

Blood samples under fasting conditions were collected during the first visit (all study participants were instructed to fast for at least 8 h) and used for the measurement of serum glucose, total cholesterol, triglyceride, high-density lipoprotein cholesterol, low-density lipoprotein cholesterol, magnesium, creatinine and cystatin C.

### 2.5. Statistical Analysis

Data are expressed as the median (25th, 75th percentile) for continuous variables and numbers (percentages) for categorical variables. The data were analyzed according to the daytime/nighttime urinary sodium excretion ratio quartiles (Q1, Q2, Q3 and Q4). Participants were divided into two groups for analysis: younger subjects (age <50 years) and older subjects (age ≥50 years). Differences among urinary sodium excretion and age groups were investigated. Specific quartiles of the daytime/nighttime urinary sodium excretion ratio for each age group were calculated, corresponding to the following values: Q1 <0.91; Q2 0.91–1.23; Q3 1.24–1.74; Q4 ≥1.75 for the age group under 50 years; and Q1 <0.83; Q2 0.83–1.10; Q3 1.11–1.46; Q4 ≥1.46 for the age group over 50 years.

The chi-square test and Wilcoxon rank-sum test were conducted to evaluate differences in categorical and continuous variables between the two age groups and to evaluate trends across the day/night ratio of urinary sodium excretion quartiles.

To explore the association between the daytime/nighttime urinary sodium excretion ratio and, the 24-h blood pressure parameters and the nocturnal dipping effect (measured as the day–night change between ABPM parameters), linear regression models were constructed. The following dependent variables were used: daytime and nighttime BP values and differences between daytime and nighttime values (i.e., nocturnal dipping) for systolic (SBP), diastolic (DBP) and mean blood pressure (MBP) and pulse pressure (PP).

The association between the daytime/nighttime urinary sodium excretion ratio and the 24-h blood pressure parameters was also explored considering daytime/nighttime urinary sodium excretion ratio quartiles, with Q1 used as the reference group. All models were adjusted for selected covariates to examine potential confounding effects. Age, gender, body mass index, smoking, use of antihypertensive medications, diuretics, previous cardiovascular diseases, dietary salt consumption, magnesium, hypercholesterolemia, diabetes and heart rate were included in the multivariate model as covariates.

Based on sodium excretion in 24-h urine, we have estimated the 24-h salt consumption using the following conversion factor: 1 mmol of sodium corresponds to 0.0584 g of salt (NaCl), previously used in large epidemiological studies [22].

All analyses were separately performed for the whole population and for both younger and older groups. The β-coefficient and confidence interval (95%, CI) relative to urinary sodium excretion quartile categories were calculated.

Statistical analyses were performed using SPSS (version 18.0, Chicago, IL, USA) and Stata statistical software (STATA) version 15.1. All statistical tests were two-sided, and the *p*-value level of significance was set at 0.05. 

## 3. Results

The characteristics of the subjects are displayed in Table 1. Results are shown for all sample subjects and quartiles of the daytime/nighttime urinary sodium excretion ratio. Differences among quartiles were also explored.

A different circadian pattern of sodium excretion was noted across quartiles of the daytime/nighttime urinary sodium excretion ratio. More specifically, subjects in the first and second quartiles could be considered low excretors of sodium during the daytime, since they excreted half the amount of sodium during the daytime than during the nighttime compared with subjects in Q4. On the other hand, subjects in Q4 could be considered high excretors of sodium during the daytime since the rate of sodium excretion during the daytime was about 40% higher than that of subjects in Q1.

However, despite these different circadian patterns of sodium excretion, 24-h urinary sodium excretion was not significantly different across quartiles (*p*-value 0.627). 

Low daytime sodium excretors were significantly older than high excretors: the median age of subjects in Q1, Q2, Q3 and Q4 was, respectively, 55, 52, 52 and 47 years, *p*-value < 0.001. Moreover, low daytime sodium excretors tended to have worse kidney function than the subjects in the highest quartiles, with higher cystatin C values (from Q1 to Q4, 0.84, 0.81, 0.80 and 0.79 mg/L respectively, *p*-value 0.023) (normal range 0.53–0.95 mg/L) and higher levels of creatinine (from Q1 to Q4, 77, 76, 72 and 71 µmol/L, respectively, *p*-value 0.001) (normal values <106 µmol/L). No significant difference in the self-reported duration of nocturnal urine collection across quartiles of the daytime/nighttime urinary sodium excretion ratio was found.

Significant differences in BP were found across quartiles of the day–night ratio of urinary sodium excretion for nighttime values. Nighttime SBP was significantly higher in Q1 and decreased across quartiles to Q4 (*p*-value ≤0.001). 

Analyzing the age groups, subjects over 50 years showed a pattern of low sodium excretors during daytime comparing with the younger group (day/night ratio of urinary sodium excretion 1.09 vs. 1.24, *p*-value ≤0.001). Despite the different circadian patterns of sodium excretion, the 24-h urinary sodium excretion was not significantly different between age groups (*p*-value 0.578). The self-reported duration of nocturnal urine collection did not vary between age groups (Supplementary Materials Table S1). As expected older subjects showed more cardiovascular risk factors (hypertension, hypercholesterolemia, diabetes) (Supplementary Materials Table S1).

Nocturnal dipping of both SBP and DBP was lower in low daytime sodium excretors (Quartile 1, respectively 8 and 8 mmHg) compared to high daytime sodium excretors (Quartile 4, respectively, 12 and 10 mmHg) (*p*-value < 0.001) (Table 1).

**Figure 1:**
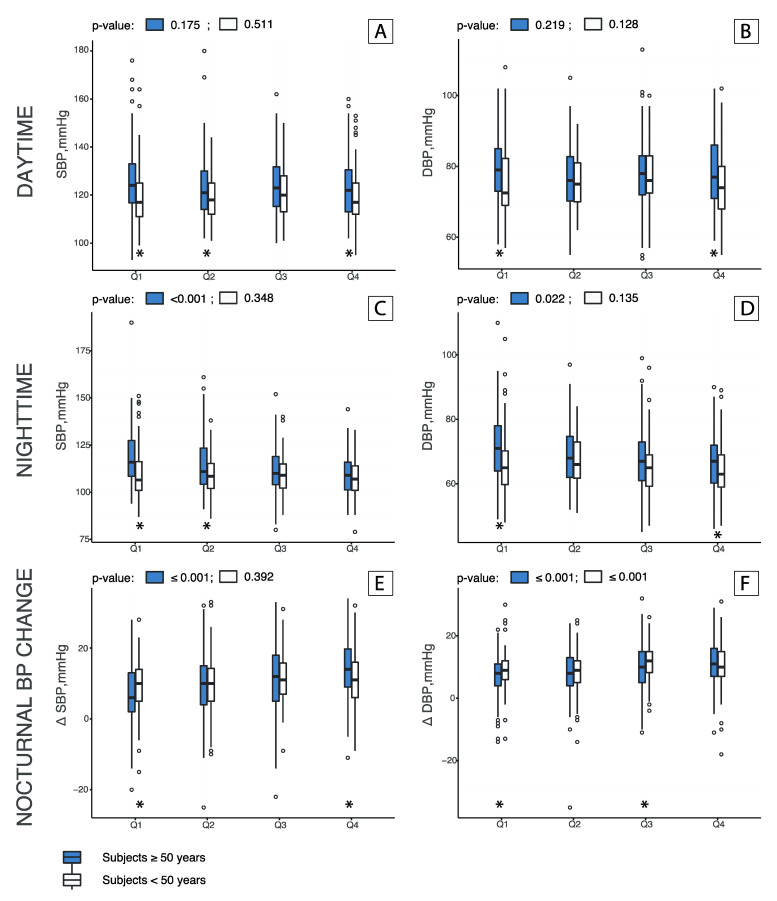
Daytime (**A**,**B**), Nighttime (**C**,**D**) and Nocturnal BP change (**E**,**F**) by age groups among quartiles of daytime/nighttime urinary sodium excretion ratio. Bars represent quartiles of the daytime/nighttime urinary sodium excretion ratio (median and confidence interval). Gray and white bars represent the age groups, respectively, of ≥50 years (589 individuals) and <50 years (473 individuals). The x axis shows quartiles (Q1, Q2, Q3 and Q4) of the daytime/nighttime urinary sodium excretion ratio. *p* values were obtained with a Wilcoxon rank-sum test performed across quartiles and among age groups. * indicate a *p*-value ≤0.05. Nighttime BP and nocturnal dipping values were significantly different across quartiles of daytime/nighttime urinary sodium excretion ratio in the older group (*p*-values for nighttime SBP ≤0.001, nighttime DBP 0.022, nocturnal SBP dipping ≤0.001 and nocturnal DBP dipping ≤0.001). No significant differences were found across quartiles for the daytime BP values in both age groups. Moreover, comparing age groups, low daytime excretors (Q1) showed significantly higher BP values in the older group comparing with the younger group for SBP, DBP, for both nighttime values and dipping values (all *p*-values < 0.05). Significant differences in nighttime MBP and nighttime PP were also found across quartiles in over 50 years (Supplementary Materials Figure S1).

The difference in BP values for the daytime, nighttime and nocturnal change was more evident in older subjects compared with younger ones. Figure 1 depicts differences across quartiles of daytime/nighttime urinary sodium excretion ratio by age group and between age groups.

We performed an analysis also considering the daytime/nighttime urinary sodium excretion ratio quartiles in order to determine which category of subjects (low or high daytime sodium excretors) had a stronger association with nighttime and nocturnal BP values.

Table 2 shows the linear associations between quartiles (Q4 reference) of daytime/nighttime urinary sodium excretion ratio and ABPM parameters (24 h, nighttime, daytime) and dipping parameters in the whole population. Results are shown in mmHg changes of BP (95% CI) across quartiles of urinary sodium excretion ratio.

Significant associations were found for nighttime BP: low daytime sodium excretor quartiles, Q1 and Q2, were associated with a significant increase in SBP and DBP, respectively, and the association was more pronounced for the lowest quartile, Q1. More specifically, Q1 for SBP and DBP showed a significant increase corresponding to (expressed in mmHg): 106.0 (86.2–125.9) *p*-value ≤0.001 and 63.2 (48.6–77.7) *p*-value ≤0.001. The same significant increase was also noted for MBP and PP for Quartile 1: 82.5 (66.8–98.2) *p*-value ≤0.001 and 42.8 (29.6–56.8) *p*-value 0.040, respectively (Supplementary Materials Table S2).

For low daytime excretors, a significantly reduced nighttime vs. daytime difference in BP values was noted for SBP and DBP in Q1: 4.1 (−11.9 to 20.1, *p*-value < 0.001) and 3.6 (−9.7 to 16.8, *p*-value < 0.001), respectively.

The association between daytime/nighttime urinary sodium excretion ratio and BP was investigated using multivariate linear regression models. Analysis by age group revealed a significant association between daytime/nighttime urinary sodium excretion ratio and nighttime BP and nocturnal dipping in the ≥50 year old age group, but not in the younger group (Supplementary Materials Table S3).

**Figure 2:**
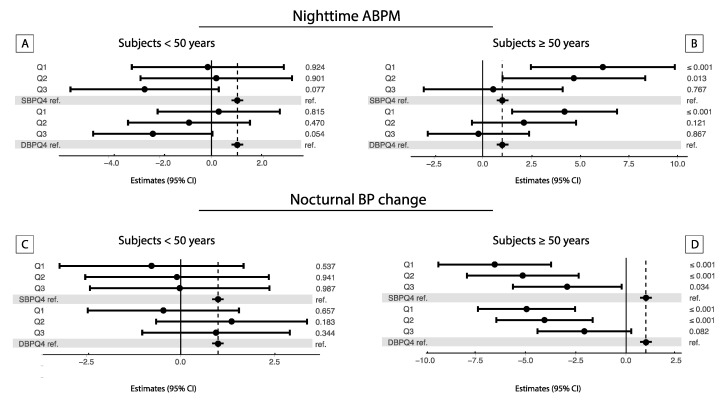
Forest plot of the linear regression analyses exploring the association between the daytime/nighttime urinary sodium excretion ratio and nighttime (**A**,**B**) and nocturnal BP changes (**C**,**D**) by age group. Analyses were performed by daytime/nighttime urinary sodium excretion ratio quartile categories, (Q4 as reference) and by age groups (≥50 years, 589 individuals and <50 years, 473 individuals). β-estimates and 95% confidence intervals were reported. Gray dotted lines represent the reference value. All models were adjusted for: age, gender, body mass index, smoking, use of antihypertensive medications, diuretics, previous cardiovascular diseases, dietary salt consumption, magnesium, hypercholesterolemia, diabetes and heart rate. Analysis for daytime are showed in Supplementary Materials Figure S2).

Figure 2 shows a forest plot of the linear association between quartiles of daytime/nighttime urinary sodium excretion ratio and daytime, nighttime and nocturnal BP changes by age group. A significant association was found only in the older group.

Low Q1 daytime excretors older than 50 years showed a significant increase in SBP and DBP for the nighttime ABPM values. The same significant association, with a reduced tendency of a BP decrease during the night, was found for the dipping values. The same tendency was observed for MBP and PP (Supplementary Materials Figure S3).

Older low daytime excretors showed a significantly reduced tendency of nocturnal dipping for all BP values (all *p*-value < 0.05). 

## 4. Discussion

The findings of the present study show that in the population older than 50 years, decreased daytime urinary sodium excretion is associated with a significant increase in nighttime BP values and with a reduced tendency to exhibit a fall in nocturnal BP. On the other hand, no associations between the circadian variation in urinary salt excretion and BP in the younger population were found.

To the best of our knowledge, this study represents the largest study conducted in a sample of the adult Caucasian population that explores the relationships between the circadian variation in urinary sodium excretion and BP. Moreover, no study has previously highlighted that the different patterns of the circadian rhythm of sodium excretion could significantly impact BP in older individuals of the population and in addition a decreased capacity to excrete sodium during daytime with increasing age.

The magnitude of this association is also relevant from a clinical perspective: older subjects present a difference of 6 mmHg for nighttime SBP between the first and fourth quartiles of the day/night ratio of the urinary sodium excretion, and the associations are independent from several possible confounding factors.

Several previous studies have shown a dose association between sodium intake and BP, and two large epidemiological studies—the INTERSALT and the PURE studies—showed a greater association in middle-aged individuals compared with younger subjects [19,23,24]. Nevertheless, several differences between the present study and previous ones have to be highlighted.

The first and most important difference consists of the method of measurement of sodium in the urine. Most previous epidemiological studies have assessed salt intake using the survey method, spot urine collection, a combination of 24-h urine and spot urine collection, or nocturnal urine collection [25,26,27,28,29], whilst very few studies have assessed salt excretion separately for daytime and nighttime. 

Here, in a large sample of the population, we performed two separate collections of urine and two distinct laboratory measurements, one for the daytime period and another one for the nighttime, in order to also investigate the role of the circadian rhythm of sodium excretion on BP.

The present findings confirm the role played by the circadian rhythm of sodium excretion in the genesis of increased nighttime BP and of the non-dipping pattern, indicating the presence of several patterns of daily sodium excretion with different impacts on BP.

Moreover, the absence in our study of a significant difference in duration of the nighttime urine collection across quartiles of urinary sodium excretion ratio, confirms that the pattern of sodium excretion was not influenced by the nighttime duration itself.

Furthermore, in our results, a wide range of variability in the circadian rhythm of sodium excretion was observed among subjects, with a different impact on BP observed in the older age group.

Subjects in Q1 and Q2 of the daytime/nighttime urinary sodium excretion ratio can be considered low daytime excretors, since more than 60% of the total amount of urinary sodium is excreted during the nighttime period, whilst subjects in Q3 and Q4 excrete about 70% of sodium during the daytime and should therefore be considered high daytime excretors.

Moreover, considering that sodium daytime excretion is one of the major determinants of the non-dipping profile [6,8,30], our findings seem to suggest that the different daily patterns of sodium excretion could help to recognize subjects at risk of nocturnal non-dipping or BP increase.

Arguably, these results could be taken into account in a clinical setting when a nocturnal BP increase or the sodium sensitivity status is under consideration, given the possibility of a partial misinterpretation of the 24-h sodium excretion in the case of a unique overnight urine collection.

Previous studies in selected populations have demonstrated that a reduced nocturnal BP fall is associated with a reduced capacity to excrete sodium during the daytime necessitating the activation of a pressure-natriuresis mechanism to facilitate the maintenance of sodium balance [11,31,32].

In a small group of subjects with salt-sensitive hypertension and a non-dipping pattern, dietary sodium restriction corresponded with the normalization of the nocturnal BP profile [33,34].

However, these studies were limited because of the well-known higher prevalence of the sodium-sensitive profile in certain populations.

Even though, because of the observational nature of our study, we cannot draw conclusions about the mechanisms of the found associations, some speculations can be made. In our study, the associations between low daytime sodium excretion and nighttime BP and a reduced nocturnal BP fall were significant only for the advanced age group but not for the younger subjects, and this aspect deserves some speculation.

We can hypothesize that the association between low daytime sodium excretion and nighttime BP could be primarily related to renal function impairment due to aging.

Indeed, the mechanisms of pressure natriuresis are substantially impaired as age increases, along with an increased prevalence of the salt-sensitive pattern and the frequency of low-renin essential hypertension [35,36].

Moreover, sodium excretion in the renal distal tubule is regulated by the influence of several hormones, among which aldosterone has one of the strongest effects [37].

Aldosterone has an activity that shows a typical circadian rhythm, characterized by inactivity during the sleeping hours [38]. This aspect could, in part, have an impact on the diurnal variation in sodium excretion, and higher aldosterone levels could result in reduced sodium excretion in the urine during the daytime and higher excretion during nighttime. The subset phenotype of low-renin hypertension, which is also associated with salt sensitivity and diuretics, is more prevalent as age increases, which could also partly contribute to our findings.

Our hypothesis that an impairment in the circadian rhythm of sodium excretion is associated with aging is also consistent with the mechanism postulated by Fukada et al., according to whom an impaired renal capacity to excrete sodium may be due to a reduced glomerular filtration rate and/or a primary increase in tubular sodium reabsorption. 

In the absence of an epidemiological and pathophysiological age threshold in the genesis of hypertension, in most guidelines age groups at risk and for interventions, are not defined homogeneously. In the present study, the population was analyzed by dividing the sample into a younger and an older group, using 50 years as the cut-off. The cut-off was decided, mainly, on the basis of three previous observations: most changes in plasma renin activity begin after 50 years, hypertensive BP patterns occur typically after this age threshold and the responsiveness to many antihypertensive drugs changes in subjects older than 50 years [39,40,41]. Furthermore, many studies used this age cut-off, which facilitates the comparability [39,42,43,44]. Not least, it is important to point out that the American Institute of Medicine, suggests lower daily salt intake for individuals older than 50 years, considered as a group at risk of greater salt sensitivity [45].

Our data indicate a renal dysfunction trend across the quartiles of daytime/nighttime urinary sodium excretion ratio when considering the significantly higher values of cystatin C in Q1 compared with subjects in Q4.

Overall, however, we are unable to discriminate with certainty whether the impaired circadian rhythm of sodium excretion is primarily the consequence of a generalized tubular dysfunction or, on the other hand, of a renal function impairment tendency (e.g., reduced nephron number with aging).

Moreover, previous studies have highlighted an inverse relationship between the renin-angiotensin system activity and serum magnesium and BP and lower level of serum magnesium with increasing age [46,47,48,49,50,51]. To note that in our study, differently from previous ones, the association found was independent from the serum magnesium levels. Moreover, previous studies have highlighted an inverse relationship between the renin-angiotensin system activity and serum magnesium and BP and lower level of serum magnesium with increasing age [46,47,48,49,50,51]. To note that in our study, differently from previous ones, the association found was independent from the serum magnesium levels.

A number of limitations in the present study should be acknowledged, too. Firstly, it is a cross-sectional study; therefore, the long-term influence of sodium intake on nighttime BP and nocturnal BP fall was not evaluated. Moreover, nighttime and daytime urine salt measurements were performed by a single 24-h urine collection, which might not be precise enough to estimate daily salt intake, considering the inter-daily variability of sodium intake and excretion. The inter-daily variability should also be considered for BP because the assessment of only one day of ABPM represents another limitation in our study. However, the single-day assessment of both BP and urinary sodium was for its technical feasibility. The assessment of such parameters over multiple consecutive days in such a large sample of subjects was impractical to realize. Moreover, the duration of the urine collection was not standardized for the daytime and nighttime period but adapted to intra-individual subject habits, and a variation in the hours of collection could affect the results. We strove to overcome this limitation by including a large number of participants, minimizing the inter-individual variability, performing urine and BP measurements on the same day and including all participants only during weekdays.

Moreover, although a single ABPM measurement might be inaccurate for its lower reproducibility, some previous studies have even highlighted a significant relationship between non-dipping BP and target organ damage using a single ABPM measurement.

Last but not least, considering the observational nature of the study design, cause-and-effect relationships cannot be postulated, and therefore, we cannot resolve a major question: does the decreased daytime sodium excretion cause an increased nighttime BP and a reduced nocturnal BP fall? Or do the higher nighttime BP values cause higher excretion of sodium during the nighttime and lower daytime excretion?

Considering the direction of the association found in previous experimental studies on sodium diet restriction and BP, we can speculate that it is the circadian rhythm of sodium excretion that influences nighttime BP. Moreover, we cannot exclude the possibility that some of the higher nighttime BP values are the consequence of poor-quality sleep due to the ABPM registration.

In conclusion, our results suggest that the pattern of sodium excretion characterized by a decrease in daytime sodium excretion is associated with a nighttime BP increase and a decreased nocturnal BP fall in older individuals.

The present findings suggest that the assessment of the individual circadian pattern of urinary sodium excretion could be useful for identifying individual variability in sodium excretory capacity, assessing salt sensitivity patterns, and potentially, detecting patients at higher risk of a nocturnal BP increase and of the non-dipping pattern.

## 5. Conclusions

The results of the present study suggest that an abnormal circadian rhythm of urinary sodium excretion may have an effect on nighttime BP in older individuals. The assessment of daytime and nighttime urinary sodium excretion in selected subjects could help in identifying an impaired circadian rhythm of sodium excretion and salt sensitivity patterns and, eventually, in detecting patients at higher risk of high nighttime BP and nocturnal non-dipping. Further prospective interventional studies could help to assess the beneficial outcome of salt intake restriction based on individualized circadian rhythms of sodium excretion.

## Figures and Tables

**Table 1:** Participant’s Characteristics (n. 1062 subjects).

	All Subjects	Quartile 1	Quartile 2	Quartile 3	Quartile 4	*p*-Value
Demographics Characteristics						
Age, Years	52 (43–60)	55 (46–67)	52 (43–61)	52 (43–60)	47 (39–55)	≤0.001
Females, *n* (%)	591 (55.7)	139 (52.3)	149 (56.02)	146 (55.30)	157 (59.02)	0.479
Body Mass Index, kg/m^2^	24.4 (22.1–27.4)	24.5 (22.0–27.8)	24.6 (22.2–27.7)	24.5 (22.7–27.4)	23.7 (21.5–26.5)	0.187
Current Smoking, *n* (%)	192 (18.2)	40 (15.2)	31 (11.7)	54 (20.6)	67 (25.3)	≤0.001
Waist/Hip, cm	0.91 (0.86–0.96)	0.91 (0.86–0.96)	0.91 (0.86–0.96)	0.91 (0.87–0.96)	0.89 (0.84–0.95)	0.075
Hypertension, *n* (%)	163 (15.5)	54 (20.5)	41 (15.5)	37 (14.2)	31 (11.7)	0.043
Diabetes, *n* (%)	22 (2.0)	6 (2.3)	6 (2.3)	6 (2.3)	4 (1.5)	0.910
Hypercolesterolemia, *n* (%)	148 (14.0)	47 (17.8)	40 (15.2)	34 (13.0)	27 (10.2)	0.080
History of CVD, *n* (%)	32 (3.0)	11(4.2)	11 (4.2)	4 (1.5)	6 (2.3)	0.184
Glycemia, mmol/L, *n* (%)	5.8 (5.5–6.2)	5.8 (5.5–6.2)	5.8 (5.5–6.2)	5.8 (5.5–6.2)	5.7 (5.4–6.2)	0.114
Cystatin C, mg/L	0.81 (0.73–0.90)	0.84 (0.75–0.94)	0.81 (0.72–0.88)	0.80 (0.73–0.90)	0.79 (0.71–0.86)	0.023
Creatinine, µmol/L	74 (64–85)	77(67–90)	76 (65–86)	72 (62–84)	71 (62–81)	≤0.001
Creatinine urine 24 h, µmol	16.4 (11.2–23.4)	15.6 (11.2–21.2)	15.5 (10.9–24.4)	16 (10.7–22.8)	17.9 (12.6–24.8)	0.050
Self-reported duration of nocturnal urine collection, hours	7.8 (7.0–8.5)	8 (7.0–8.8)	7.8 (7.0–8.5)	7.6 (7.0–8.5)	8 (7.0–8.8)	0.260
Day/night ratio of urinary sodium excretion, mmol	1.15 (0.85–1.57)	0.68 (0.56–0.77)	0.99 (0.93–1.08)	1.33 (1.24–1.44)	2.05 (1.77–2.47)	<0.001
24-h Urinary sodium excretion, mmol	163.5 (114–228)	133.2 (92.6–173.8)	134.6 (96.5–177.1)	131.5 (95.8–169.3)	125.6 (92.1–163.5)	0.627
Daytime Urinary sodium excretion, mmol	57.6 (40.7–83.2)	51.8 (35.6–67.6)	67.2 (48.6–88.9)	75.9 (54.6–97.4)	87.5 (63.4–111.3)	≤0.001
Nighttime Urinary sodium excretion, mmol	67.7 (48.7–92.6)	81.2 (56.6–104.9)	68 (48.8–90.2)	55.1 (41.6–72.4)	38.9 (27.8–55.3)	≤0.001
24-Hours ABPM						
24-h SBP, mmHg	117 (111–126)	119 (112–129)	117 (111–126)	119 (112–126)	116 (110–123)	≤0.001
24 h DBP, mmHg	73 (68–80)	74 (69–81)	73 (68–79)	74 (69–80)	72 (67–79)	0.051
Heart Rate 24 h	70 (64–75)	70 (63–75)	70 (65–75)	71 (64–75)	70 (65–75)	0.561
24-PP, mmHg	44 (39–49)	44 (39–51)	44 (40–49)	44 (40–49)	43 (39–47)	0.240
Daytime ABPM						
Daytime SBP, mmHg	121 (114–128)	122 (114–130)	120 (113–128)	122 (114–130)	119 (113–126)	0.224
Daytime DBP, mmHg	76 (70–83)	77 (71–84)	75 (70–82)	77 (72–83)	75 (69–82)	0.200
Heart Rate day-time	73 (67–79)	72 (65–79)	73 (67–78)	73 (67–79)	73 (68–78)	0.780
Daytime PP, mmHg	44 (39–50)	44 (39–50)	44 (40–50)	44 (39–49)	43 (39–48)	0.406
Nighttime ABPM						
Nighttime SBP, mmHg	110 (103–118)	114 (104–124)	110 (103–119)	110 (103–117)	108 (101–115)	≤0.001
Nighttime DBP, mmHg	66 (61–73)	68 (61–77)	67 (62–74)	66 (60–72)	65 (59–71)	0.013
Heart Rate night-time	62 (56–68)	61 (56–67)	62 (57–67)	63 (57–68)	62 (56–68)	0.758
Nighttime PP, mmHg	43 (39–48)	43 (39–51)	43(38–48)	44 (40–48)	42 (38–47)	≤0.001
Nocturnal BP Change						
Difference SBP day-night, mmHg	10 (5–16)	8 (3–13)	10 (5–15)	12 (6–18)	12 (6–17)	≤0.001
Difference DBP day-night, mmHg	10 (5–14)	8 (5–12)	9 (4–12)	11 (7–15)	10 (7–15)	≤0.001
Difference PP day-night, mmHg	1 (−3–5)	0 (−3–4)	1 (−2–5)	1 (−3–5)	1 (−2–4.5)	0.474

Data are shown according to daytime/nighttime urinary sodium excretion ratio quartiles (Q1, Q2, Q3, Q4). CVD: cardiovascular disease.

**Table 2:** Association between ambulatory blood pressure parameters (24-h, daytime and nighttime), nocturnal change and daytime/nighttime urinary sodium excretion ratio by quartiles.

Overall Population				
Urinary Sodium Excretion Ratio	SBP, mmHg (95%CI)	*p*-Value	DBP, mmHg (95%CI)	*p*-Value
24 Hours ABPM				
Q1	113.5 (94.3–132.7)	0.206	66.7 (53.0–80.4)	0.583
Q2	112.1 (95.1–129.1)	0.492	66.3 (54.1–78.4)	0.736
Q3	111.4 (96.4–126.3)	0.897	66.0 (55.3–76.7)	0.965
Q4	Reference		Reference	
Daytime ABPM				
Q1	116.9 (96.7–137.1)	0.841	68.8 (54.2–83.4)	0.484
Q2	116.6 (98.6–134.2)	0.974	69.4 (56.5–82.3)	0.530
Q3	116.7 (100.9–132.4)	0.952	69.9 (58.5–81.2)	0.903
Q4	Reference		Reference	
Nighttime ABPM				
Q1	106.0 (86.2–125.9)	≤0.001	63.2 (48.6–77.7)	<0.001
Q2	101.6 (84.1–119.2)	0.021	60.5 (47.6–73.3)	0.018
Q3	99.0 (83.7–114.4)	0.547	58.5 (47.8–69.8)	0.889
Q4	Reference		Reference	
Nocturnal BP change				
Q1	4.1 (−11.9, −20.1)	≤0.001	3.6 (−9.7–16.8)	≤0.001
Q2	8.3 (−5.9,−22.5)	≤0.001	−4.9 (−4.8–18.7)	≤0.001
Q3	10.6 (−1.9–23.1)	0.498	9.25 (−1.1–19.6)	0.710
Q4	Reference		Reference	

All models adjusted for: age, gender, body mass index, smoking, use of antihypertensive medications, diuretics, previous cardiovascular diseases, dietary salt consumption, magnesium, hypercholesterolemia, diabetes and heart rate.

## Supplementary Materials

The following are available online at https://www.mdpi.com/2072-6643/12/7/2013/s1, Table S1: Participant’s characteristics by age groups, Table S2: Association between MBP, PP (24-h, daytime and nighttime, nocturnal change) and daytime/nighttime urinary sodium excretion ratio by quartiles, Table S3: Multiple Regression models exploring the association between daytime/nighttime urinary sodium excretion ratio and 24-h blood pressure parameters and nocturnal BP changes in the overall population and by age groups, Figure S1: MBP and PP (daytime, nighttime and nocturnal change) by age groups among quartiles of daytime/nighttime urinary sodium excretion ratio, Figure S2: Forest plot of the linear regression analyses exploring the association between the daytime/nighttime urinary sodium excretion ratio and daytime BP by age groups, Figure S3: Forest plot of the linear regression analyses exploring the association between the daytime/nighttime urinary sodium excretion ratio and MBP and PP (daytime, nighttime and nocturnal changes) by age groups.

## Abbreviations

SBP	Systolic Blood Pressure
DBP	Diastolic Blood Pressure
MBP	Mean Blood Pressure
PP	Pulse Pressure
ABPM	Ambulatory Blood Pressure Monitoring
BP	Blood Pressure
CVD	Cardio Vascular Diseases
