# Supplementary material for: Impaired Daytime Urinary Sodium Excretion Impacts Nighttime Blood Pressure and Nocturnal Dipping at Older Ages in the General Population
Source: Nutrients. 2020 Jul 7;12(7):2013. doi: 10.3390/nu12072013 (PMC7400814; doi:10.3390/nu12072013)

Supplementary Table 1. Participants characteristics by age groups

	Younger Group (<50 years old) (n. 473)	Older Group (≥50 years old) (n. 589)	p-value
**Demographics Characteristics**			
Age, years	41 (34-46)	59 (54-67)	≤0.001
Females, n (%)	273 (57.7)	318 (53.9)	0.224
Body Mass Index, kg/m^2	23.6 (21.5-26.2)	25 (22.7-28.2)	≤0.001
Current smoking, n (%)	82 (17.5)	110 (18.7)	0.610
Waist/Hip, cm	0.89 (0.84-0.93)	0.92 (0.88-0.97)	≤0.001
Hypertension, n (%)	12 (2.5)	151 (26.0)	≤0.001
Diabetes, n (%)	1 (0.21)	21 (3.6)	≤0.001
Hypercholesterolemia, n (%)	13 (2.8)	135 (23.3)	≤0.001
Hystory of CVD, n (%)	1 (0.21)	31 (5.4)	≤0.001
Glycemia, mmol/l, n (%)	5.7 (5.4-6)	6 (5.7-6.5)	≤0.001
Cystatin C, mg/L	0.77 (0.70-0.84)	0.85 (0.77-0.95)	≤0.001
Creatinine, µmol/L	73 (61-85)	75 (66-86)	0.121
Creatinine urine 24 hour, µmol	17.9 (12.2-24.1)	15.2 (10.5-22.8)	0.001
Self-reported duration of nocturnal urine collection (hours)	8 (7-9)	7.5 (7.0-8.5)	0.066
Day/night ratio of urinary sodium excretion	1.24 (0.91-1.76)	1.09 (0.81-1.45)	≤0.001
24-hours Urinary sodium excretion, mmol	165 (116-234)	161 (113-226)	0.578
Daytime Urinary sodium excretion, mmol	72.4 (50.4-99.5)	65.6 (46.4-87.6)	0.002
Nighttime Urinary sodium excretion, mmol	55.8 (39.6-80.1)	60.1 (42.3-84.0)	0.056
**24-Hours ABPM**			
24-hour SBP, mmHg	116 (110 -122)	120 (112 -128)	≤0.001
24 hour DBP, mmHg	72 (67-78)	75 (69 -81)	≤0.001
Heart Rate 24 hour	71 (65-76)	69 (64-74)	0.007
24-MBP, mmHg	92 (87-98)	96 (90-102)	≤0.001
24-PP, mmHg	43 (39-47)	44 (40-50)	0.007
**Daytime ABPM**			
Daytime SBP, mmHg	118 (112-126)	123 (115-132)	≤0.001

Daytime DBP, mmHg	75 (70 – 81)	78 (71-84)	≤0.001
Heart Rate day-time	74 (68 -80)	72 (67-78)	0.014
Daytime MBP, mmHg	95 (89-101)	98 (92-105)	≤0.001
Daytime PP, mmHg	43 (39 -48)	45 (40-51)	≤0.001
**Nighttime ABPM**			
Nighttime SBP, mmHg	108 (101.5-115)	112 (105-121.5)	≤0.001
Nighttime DBP, mmHg	65 (59.5-70.5)	68 (62-75)	≤0.001
Heart Rate night-time	62 (56-68)	62 (57-67)	0.215
Nighttime MBP, mmHg	84 (79-91)	88 (82 -96)	≤0.001
Nighttime PP, mmHg	43 (39 -47)	43 (39-50)	0.677
**Nocturnal BP Change**			
Difference SBP day-night, mmHg	10 (5.5-15)	11 (4-16)	0.552
Difference DBP day-night, mmHg	10 (7-14)	9 (5-14)	0.118
Difference PP day-night, mmHg	0 (3-4)	1 (2.5- 5)	0.300
Difference MBP day-night, mmHg	10 (7-14)	9 (5-159	0.075

Supplementary Table 2

Overall Population				
**Urinary Sodium Excretion Ratio**	**MBP MmHg (95%CI)**	**p-value**	**PP MmHg (95%CI)**	**p-value**
**24 Hours ABPM**				
**Q1**	88.1 (73.1-103.1)	0.306	47.3 (33.7-60.9)	0.214
**Q2**	87.2 (73.9-100.5)	0.597	46.3 (34.3-58.4)	0.549
**Q3**	86.8 (75.1-98.4)	0.890	45.9 (35.3-56.5)	0.882
**Q4**	Reference		Reference	
**Daytime ABPM**				
**Q1**	91.2 (75.3-106.7)	0.822	48.3 (33.6-62.9)	0.322
**Q2**	91.4 (77.3-105.2)	0.762	74.4 (34.5-60.4)	0.562
**Q3**	91.7 (79.3-103.)	0.984	46.9 (36.6-58.3)	0.873
**Q4**	Reference		Reference	
**Nighttime ABPM**				
**Q1**	82.5 (66.8-98.2)	≤0.001	42.8 (29.6-56.8)	0.040
**Q2**	78.9 (65.1-92.9)	0.013	41.1 (28.8-53.4)	0.405
**Q3**	76.8 (64.6-88.9)	0.775	40.5 (29.7-51.2)	0.297
**Q4**	Reference		Reference	
**Nocturnal BP change**				
**Q1**	4.3 (-9.1,-17.7)	≤0.001	0.68 (-10.7 -11.9)	0.176
**Q2**	8.1 (-3.8, -19.9)	≤0.001	1.6 (-8.6-11.6)	0.996
**Q3**	10.4 (-0.01-20.8)	0.858	1.57 (-7.33 -10.3)	0.168
**Q4**	Reference		Reference	

All Models adjusted for: age, gender, body mass index, smoking, use of antihypertensive medications, previous cardiovascular diseases, dietary salt consumption, magnesium, hypercholesterolemia, diabetes and heart rate.

Supplementary Table 3. Multiple Regression models exploring the association between daytime/nighttime urinary sodium excretion ratio and 24-hour blood pressure parameters and nocturnal BP changes in the overall population and by age groups.

	Overall Population		Younger Group (≤50 years old)		Older Group (≥50 years old)	
**ABPM Parameters**	**β-coefficient (95%, confidence interval)**	**p-value**	**β-coefficient (95%, confidence interval)**	**p-value**	**β-coefficient (95%, confidence interval)**	**p-value**
**24-Hour SBP**	-0.710 (-1.773-0.352)	0.190	-0.753 (-1.998-0.492)	0.235	-0.812 (-2.525-0.902)	0.352
**24-Hour DBP**	-0.570 (-1.340-0.199)	0.146	-0.965 (-1.945-0.015)	0.054	-0.281 (-1.484-0.923	0.647
**24-Hour MBP**	-0.627 (-1.461-0.206)	0.140	-0.839 (-1.877-0.198)	0.113	-0.546 (-1.871-0.779)	0.418
**24-Hour PP**	-0.123 (-0.879-0.634)	0.750	0.227 (-0.558-1.013)	0.569	-0.516 (-1.738-0.707)	0.408
**Daytime SBP**	-0.125 (-1.246-0.995)	0.826	-0.653 (-1.953-0.648)	0.324	0.265 (-1.552-2.082)	0.774
**Daytime DBP**	-0.076 (-0.899-0.747)	0.855	-0.825 (-1.851-0.201)	0.115	0.585 (-0.708-1.878)	0.374
**Daytime MBP**	-0.109 (-0.990-0.771)	0.807	-0.736 (-1.809-0.337)	0.178	0.409 (-0.999-1.817)	0.569
**Daytime PP**	0.027(-0.841-0.786)	0.947	0.170 (-0.709-1.049)	0.704	-0.280 (-1.594-1.034)	0.675
**Nighttime SBP**	-2.078 (-3.197- -0.958)	**<0.001**	-1.011 (-2.323-0.300)	0.130	-3.346 (-5.118,-1.574)	**<0.001**
**Nighttime DBP**	-1.521(-2.344,-0.696)	**<0.001**	-1.040 (-2.093-0.012)	0.053	-2.122 (-3.417,-0.827)	**0.001**
**Nighttime MBP**	-1.783 (-2.670,-0.896)	**<0.001**	-1.008 (-2.106-0.090)	0.072	-2.732 (-4.134,-1.329)	**<0.001**
**Nighttime PP**	-0.569 (-1.362-0.223)	0.159	0.078 (-0.805-0.962)	0.861	-1.303(-2.521-,0.086)	**0.036**
**Nocturnal BP change**						
**SBP**	2.152 (1.270-3.034)	**<0.001**	0.436 (-0.647-1.520)	0.429	4.100 (2.720-4.480)	**<0.001**
**DBP**	1.610 (0.874 -2.346)	**<0.001**	0.328 (-0.579-1.235)	0.477	2.992 (1.828-4.157)	**<0.001**
**MBP**	1.851 (1.111-2.590)	**<0.001**	0.363 (-0.532-1.259)	0.426	3.520 (2.353-4.688)	**<0.001**
**PP**	0.577 (0.049-1.204)	0.071	0.053 (-0.795-0.902)	0.902	1.232 (0.287-2.177)	**0.011**

All Models adjusted for: age, gender, body mass index, smoking, use of antihypertensive medications, previous cardiovascular diseases, dietary salt consumption, magnesium, hypercholesterolemia, diabetes and heart rate.

Supplementary Figure 1

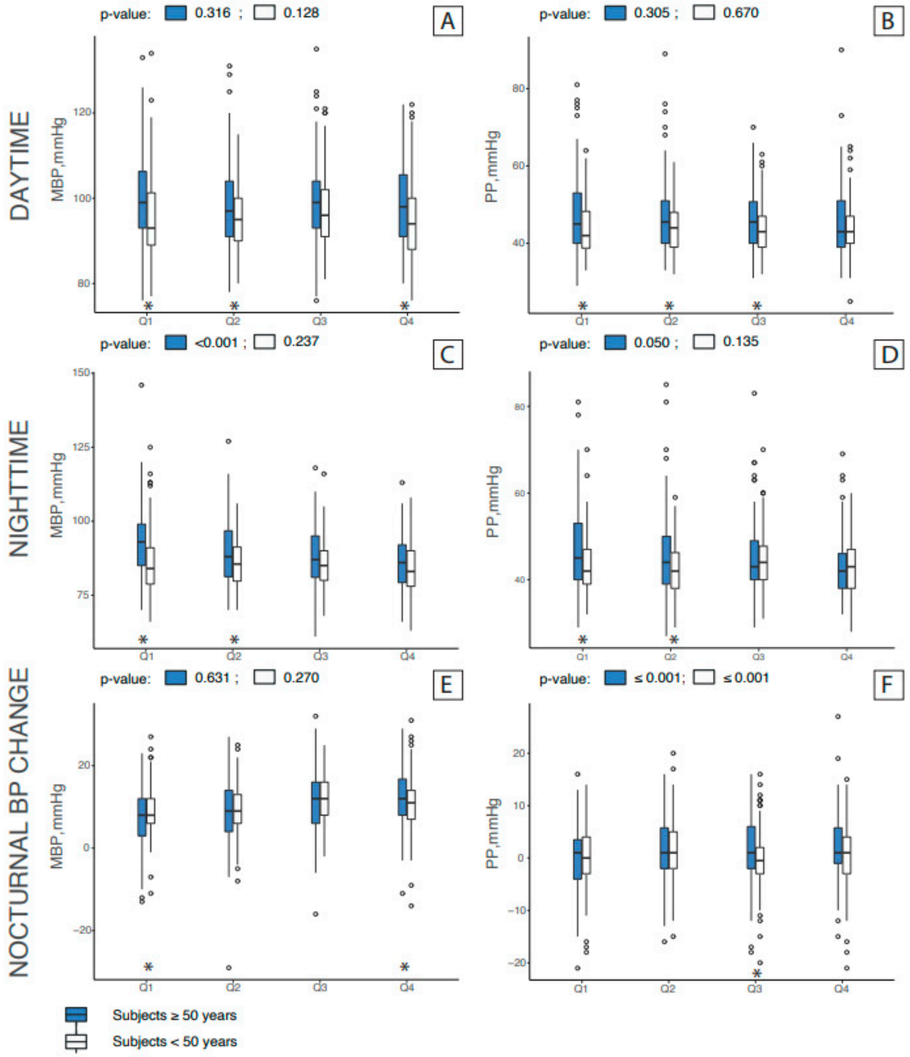

Supplementary Figure 2

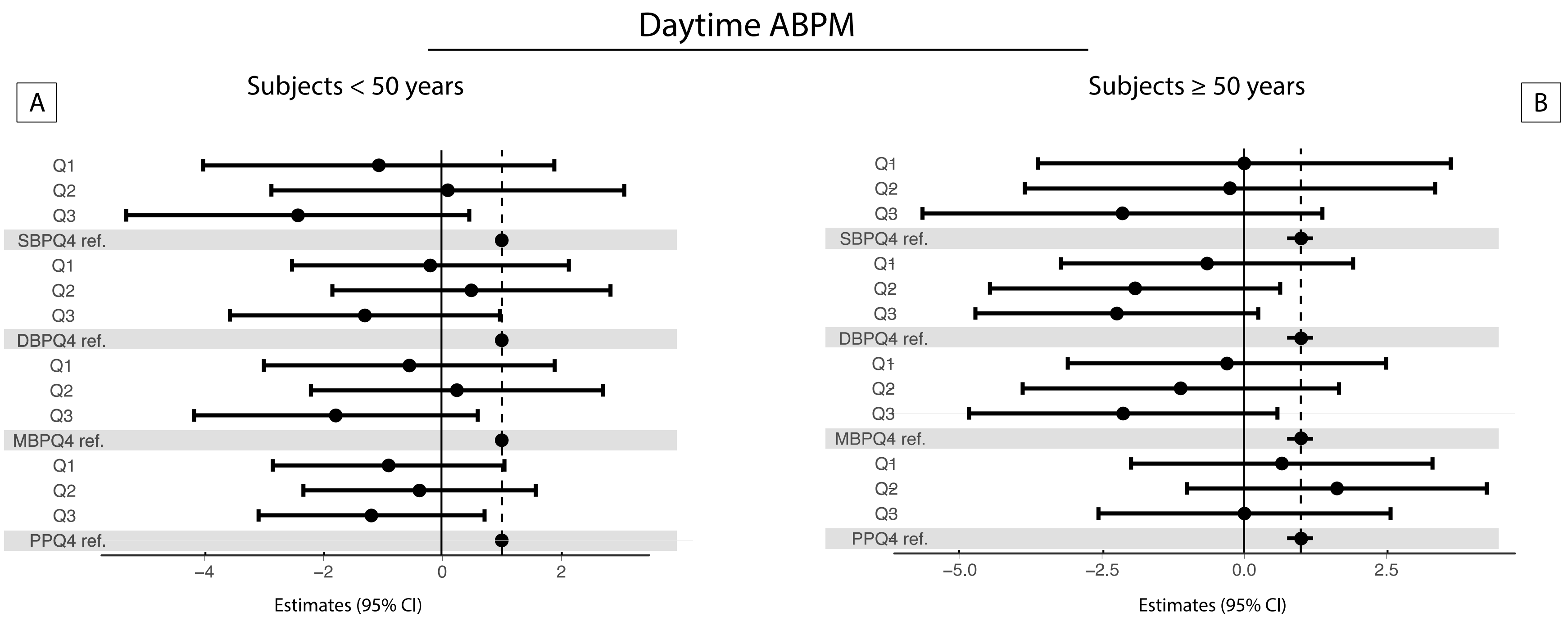

Supplementary Figure 3

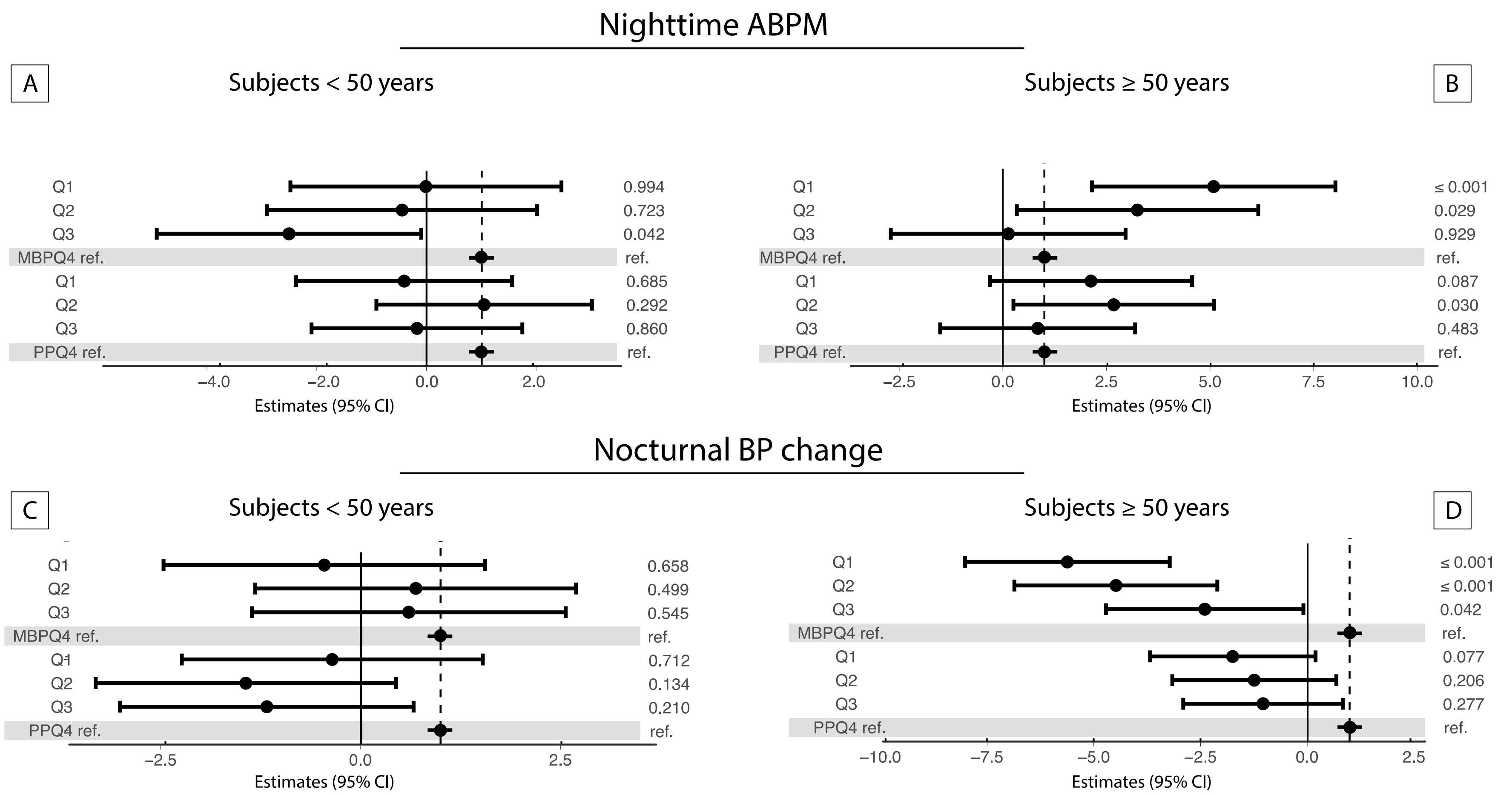

Supplement: Supplementary file 1 [file nutrients-12-02013-s001.pdf]
